# Beyond mean heart dose: quantitative impact of sub-structure spatial dosimetrics on subclinical myocardial strain in thoracic radiotherapy

**DOI:** 10.3389/fcvm.2026.1884120

**Published:** 2026-08-06

**Authors:** Zhang Yanjun, Xiaochen Han, Feifei Wang, Ruoling Han, Yasi Zhang, Ziqi Zhao, Dan Liu, Zifeng Chi

**Affiliations:** Fourth Hospital of Hebei Medical University, Shijiazhuang, China

**Keywords:** acute subclinical strain alteration, cardiac substructures, dose-volume effect, radiation cardio-oncology, two-dimensional speckle-tracking echocardiography

## Abstract

**Purpose:**

To evaluate the spatial dosimetric relationships between cardiac substructures and hyper-acute subclinical myocardial strain alterations after thoracic intensity-modulated radiotherapy (IMRT).

**Methods:**

Forty patients with thoracic malignancies were prospectively enrolled. Two-dimensional speckle-tracking echocardiography (2D-STE) was performed at baseline and 1–2 days post-radiotherapy to assess left ventricular global longitudinal strain (LVGLS) and right ventricular free wall longitudinal strain (RVFWLS). Cardiac substructures, including the atrioventricular node (AVN), sinoatrial node (SAN), and right coronary artery (RCA), were contoured standardized. Acute subclinical strain alteration was defined as a ≥ 15% relative reduction from baseline. Optimal dosimetric cutoffs were determined via Maxstat analysis, and independent predictors were identified using multivariable logistic regression.

**Results:**

Immediately post-radiotherapy, the incidence of hyper-acute subclinical strain alterations was 65% (26/40) for the right ventricle and 40% (16/40) for the left ventricle, captured by 2D-STE prior to any conventional LVEF decline. Multivariable analysis identified AVN *D*_mean_ ≥ 6.83 Gy as an independent predictor of acute LV strain alterations (OR = 4.49, 95%CI: 3.46–5.82, *P* < 0.001). For the right ventricle, RCA *V*_5_ ≥ 77.56% (OR = 6.0, 95%CI: 4.26–8.53, *P* < 0.001), SAN *D*_max_ ≥ 33.36 Gy (OR = 3.07, 95%CI: 2.11–4.44, *P* < 0.001) were independent predictors.

**Conclusion:**

Hyper-acute subclinical strain alterations are highly prevalent immediately after thoracic radiotherapy, with right-sided structures exhibiting enhanced radiosensitivity. Dosimetric metrics of the AVN, SAN, and RCA serve as independent predictors. Although these data-driven cutoffs are exploratory and hypothesis-generating, they suggest that independent sparing of the conduction system and coronary vasculature should be considered in precision radiotherapy, warranting validation in future large-scale, multicenter trials.

## Introduction

With advancing multimodal cancer therapies and prolonged patient survival, radiation-induced heart disease (RIHD) has emerged as a major non-oncological determinant of long-term prognosis. Given its clear dose-dependency, RIHD represents a preventable toxicity via radiotherapy planning optimization (1, 2). Current guidelines from the European Society of Cardiology (ESC) and Chinese Anti-Cancer Association highlight that a ≥ 15% relative decline in left ventricular global longitudinal strain (LVGLS) or right ventricular free wall longitudinal strain (RVFWLS) measured by two-dimensional speckle-tracking echocardiography (2D-STE) is the most sensitive biomechanical marker for acute subclinical cancer therapeutics-related cardiac dysfunction (CTRCD), outperforming conventional left ventricular ejection fraction (LVEF) (3–5).

However, traditional radiobiological models conceptualize the heart as a single parallel organ, relying on mean heart dose (MHD) for constraint compliance. This approach overlooks the profound radiosensitivity heterogeneity among cardiac substructures and the highly non-uniform dose distributions characteristic of modern intensity-modulated radiotherapy (IMRT). In the era of precision oncology, restricting evaluation to MHD is insufficient. Characterizing the spatial dose-volume effects on distinct cardiac substructures—particularly the electrical conduction nodes (sinoatrial node SAN and atrioventricular node AVN) (6) and the macrovascular vasculature (right coronary artery RCA) (6–9)—has become a pivotal frontier.

Prospective data linking substructure dosimetry to acute myocardial biomechanical strain remain sparse. Specifically, the right-sided cardiac system, characterized by a thin-walled and low-pressure anatomy, may exhibit enhanced acute radiosensitivity; yet definitive clinical dose constraints for these sub-structures are not established (9, 10). Therefore, this prospective study utilized 2D-STE as a hyper-early surveillance tool to quantify the relationship between sub-structure dosimetric parameters and acute subclinical strain alterations after thoracic IMRT. Our objective was to identify radiosensitive sub-structures and generate exploratory, hypothesis-generating thresholds to provide initial evidence for personalized sub-structure sparing.

## Materials and methods

### Patient selection

This single-center prospective study enrolled 40 consecutive patients with thoracic malignancies (including lung, esophageal, and breast cancers) treated with IMRT between May 2021 and May 2023. Inclusion criteria were age ≥ 18 years and preserved baseline left ventricular ejection fraction LVEF > 50%. Patients with prior cardiovascular disease, previous thoracic irradiation, or suboptimal echocardiographic image quality were excluded. The trial was approved by the Institutional Ethics Committee, and all participants provided written informed consent (Table 1).

**Table 1:** Patient clinical characteristics.

Characteristic	Category	No. of patients (%)
Sex	Male	14 (35%)
	Female	26 (65%)
Age (years)	≤60	20 (50%)
	>60	20 (50%)
Tumor type	Lung cancer	18 (45%)
	Esophageal cancer	12 (30%)
	Breast cancer	10 (25%)
Framingham risk score	≤6分	8 (20%)
	7–10分	12 (30%)
	11–15分	10 (25%)
	>15分	10 (25%)
Treatment regimen	Radiotherapy (RT)	16 (40%)
	Immunotherapy + RT	9 (22.5%)
	Chemotherapy + RT	15 (37.5%)

### Radiotherapy and substructure dosimetry

All patients underwent CT-guided IMRT. According to consensus atlases and anatomical landmarks (11), the whole heart, chambers (LV, RV, LA, RA), left anterior descending artery (LAD), right coronary artery (RCA), sinoatrial node (SAN), and atrioventricular node (AVN) were standardized contoured (12, 13). Dosimetric metrics, including mean dose (*D*_mean_), maximum dose (*D*_max_), and volume percentages receiving ≥5, 10, 20, 30, and 40 Gy (*V*_5_- *V*_40_), were retrieved from the treatment planning system.

### Assessment of acute subclinical myocardial strain alterations

Standard apical views were acquired pre-radiotherapy and 1–2 days post-radiotherapy. Left ventricular global longitudinal strain (LVGLS), layer-specific strain (GLSendo, GLSmid, GLSepi), and right ventricular free wall longitudinal strain (RVFWLS) were calculated offline via EchoPAC software. Under preserved conventional LVEF, an acute subclinical LV or RV strain alteration was defined as a ≥ 15% relative reduction in LVGLS or RVFWLS from baseline, respectively (Table 2).

**Table 2:** Comparison of baseline characteristics by different treatment modality.

Parameters	RT	Chemotherapy + RT	Immunotherapy + RT	*P*
Age (years)	66.0 ± 9.20	54.38 ± 10.65	59.25 ± 9.03	0.08
Framingham risk score	15.45 ± 4.62	7.13 ± 4.34	14.73 ± 4.03	0.07
LVEF (%)	64.00 ± 3.71	65.13 ± 4.29	65.25 ± 1.89	0.62
LVFS (%)	35.25 ± 3.15	37.00 ± 2.88	35.00 ± 0.82	0.23
MAPSE (mm)	16.00 ± 2.33	15.13 ± 1.13	15.75 ± 1.50	0.73
E_M_ (m/s)	0.71 ± 0.10	0.71 ± 0.19	0.72 ± 0.090	0.96
A_M_ (m/s)	0.96 ± 0.15	0.73 ± 0.10	0.80 ± 0.27	0.12
E_M_/A_M_	0.76 ± 0.22	0.91 ± 0.33	0.99 ± 0.34	0.55
s’_M_ (m/s)	0.09 ± 0.03	0.09 ± 0.02	0.10 ± 0.01	0.34
e’_M_ (m/s)	0.08 ± 0.02	0.09 ± 0.01	0.13 ± 0.02	0.25
E_M_/e’_M_	9.63 ± 1.41	7.13 ± 1.55	5.75 ± 0.96	0.72
LVGLS-Endo (%)	−24.01 ± 3.21	−24.99 ± 4.41	−25.40 ± 2.96	0.92
LVGLS-Epi (%)	−18.80 ± 3.03	−19.34 ± 3.64	−19.85 ± 2.83	0.84
LVGLS-mid (%)	−21.18 ± 3.11	−21.96 ± 3.97	−22.40 ± 2.85	0.90
E_T_	0.55 ± 0.12	0.59 ± 0.08	0.58 ± 0.13	0.56
E_T_/A_T_	1.21 ± 0.33	1.07 ± 0.32	1.31 ± 0.54	0.56
s'_T_	0.14 ± 0.03	0.13 ± 0.03	0.13 ± 0.02	0.68
e'_T_	0.10 ± 0.03	0.10 ± 0.02	0.14 ± 0.02	0.13
E_T_/e'_T_	4.88 ± 1.46	6.13 ± 1.36	4.25 ± 0.96	0.07
DT	173.5 ± 38.21	200.9 ± 60.47	141.5 ± 75.19	0.39
FAC	0.35 ± 0.10	0.40 ± 0.10	0.34 ± 0.04	0.20
TAPSE	20.75 ± 3.45	22.25 ± 3.11	18.75 ± 2.75	0.16
RVFWLS	−20.13 ± 7.43	−22.38 ± 7.89	−23.25 ± 4.86	0.68

### Statistical analysis

Data were analyzed using SPSS 27.0 and R 4.3.1, with non-normally distributed metrics presented as medians (interquartile ranges). Continuous dosimetric parameters were dichotomized into binary predictors using the Maxstat algorithm based on strain alteration outcomes. Univariable screening was set at *P* < 0.10, and structurally redundant variables were excluded via a Spearman correlation matrix (threshold *r* > 0.70). Under strict events-per-variable constraints (EPV≥10; 16 LV events permitting 1 variable, 26 RV events permitting 2 variables), automated stepwise selection was eschewed; instead, mechanistic core parameters (AVN *D*_mean_ for the LV model; SAN *D*_max_ and RCA *V*_5_ for the RV model) were manually entered into the final multivariable logistic regression. All derived thresholds must be interpreted strictly as exploratory and hypothesis-generating. Two-tailed *P* < 0.05 indicated statistical significance.

## Results

### Baseline and dosimetric characteristics

**Figure 1:**
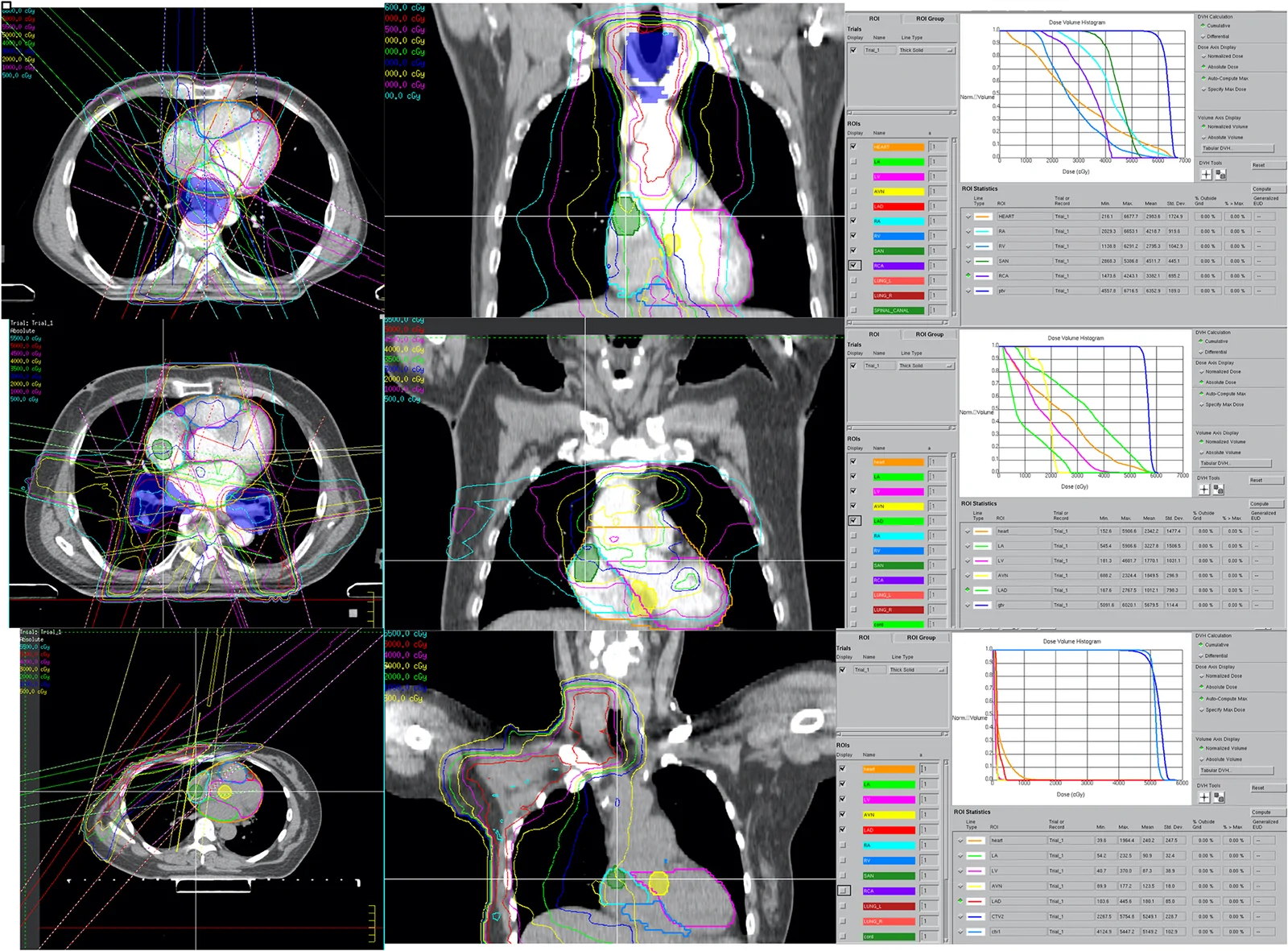
2D isodose distribution and DVH of right heart.

**Figure 2:**
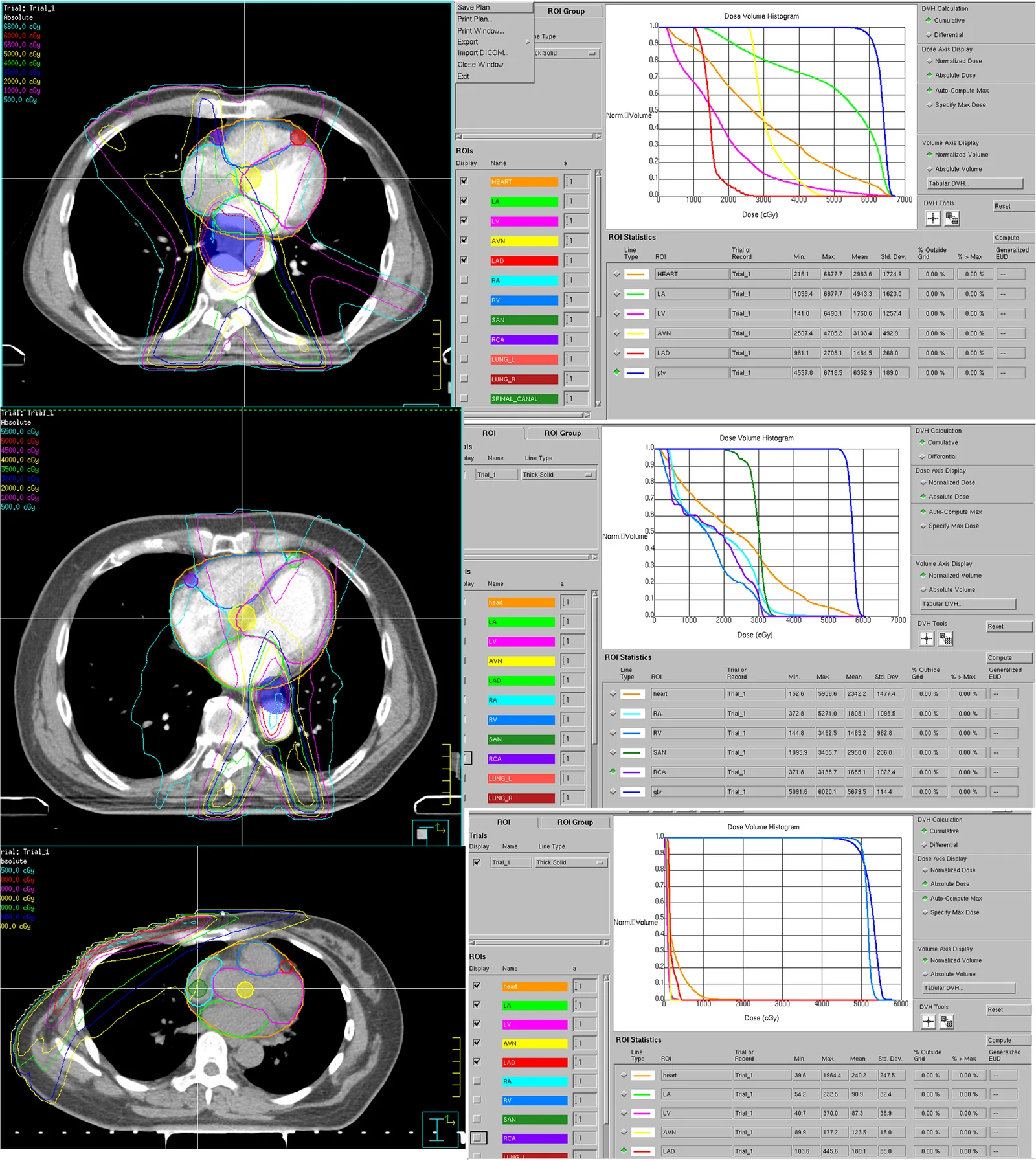
2D isodose distribution and DVH of left heart.

Forty patients (18 lung, 12 esophageal, and 10 breast cancers) were evaluated; baseline traits and cardiac functions were well-balanced across groups (all *P* > 0.05). Dosimetric analysis revealed pronounced spatial heterogeneity among cardiac substructures (Table 3). The median mean heart dose (MHD) was 17.84 Gy. For conduction structures, high exposure was observed: the atrioventricular node (AVN) showed a median *D*_mean_ of 12.1 Gy (IQR: 1.28–23.26 Gy; *D*_max_ 17.81 Gy), and the sinoatrial node (SAN) showed a median *D*_mean_ of 12.54 Gy (IQR: 2.42–26.25 Gy; *D*_max_ 24.19 Gy) with a median *V*_5_ of 95.55%. For vascular structures, the right coronary artery (RCA) was widely exposed to low doses, with a median *V*_5_ of 71.67% (IQR: 47.64%–100%). These findings indicate that specific substructures face extensive low-dose exposure despite well-controlled overall heart metrics (see Table 4; Figures 1, 2).

**Table 3:** Comparison of echocardiographic parameters before and after radiotherapy.

Echocardiographic parameters	Pre-radiotherapy	Post-radiotherapy	*P*
LVEF (%)	64.70 ± 3.57	62.80 ± 5.74	0.11
LVFS (%)	35.9 ± 2.77	34.2 ± 3.82	0.12
MAPSE (mm)	17.02 ± 1.52	15.71 ± 1.78	0.006
E_M_ (m/s)	0.70 ± 0.134	0.61 ± 0.10	0.02
A_M_ (m/s)	0.83 ± 0.19	0.88 ± 0.20	0.42
E_M_/A_M_	0.86 ± 0.29	0.73 ± 0.21	0.09
s’_M_ (m/s)	0.09 ± 0.02	0.09 ± 0.03	0.79
e’_M_ (m/s)	0.09 ± 0.03	0.09 ± 0.02	0.95
E_M_/e’_M_	7.85 ± 2.06	7.05 ± 1.43	0.16
LVGLS-Endo (%)	−24.68 ± 3.56	−18.8 ± 3.12	<0.0001
LVGLS-Epi (%)	−19.23 ± 3.12	−14.4 ± 2.36	<0.0001
LVGLS-mid (%)	−21.74 ± 3.3	−16.41 ± 2.69	<0.0001
E_T_	0.57 ± 0.11	0.49 ± 0.11	0.12
E_T_/A_T_	1.17 ± 0.37	0.88 ± 0.25	0.02
S'_T_	0.13 ± 0.03	0.13 ± 0.03	0.47
e'_T_	0.11 ± 0.03	0.09 ± 0.02	0.04
E_T_/e'_T_	5.25 ± 1.48	5.60 ± 1.57	0.76
D_T_	178.05 ± 57.33	169.35 ± 34.28	0.33
FAC	0.36 ± 0.10	0.35 ± 0.09	0.87
TAPSE	20.95 ± 3.3	20.40 ± 3.46	0.56
RVFWLS	−21.65 ± 6.98	−21.35 ± 5.75	0.65

**Table 4:** Dose distribution of the heart and cardiac substructures.

substructures	Dmean (Gy)	Dmax (Gy)	V5 (%)	V10 (%)	V20 (%)	V30 (%)	V40 (%)
Heart	17.84 (2.35,23.42)	57.73 (19.64,64.54)	66.99 (14.53,90.4)	60.13 (2.21,73.11)	34.26 (0,48.31)	21.11 (0,33.13)	9.76 (0,17.79)
LV	12.62 (1.16,17.78)	40.39 (4.0,55.3)	70.5 (0.79,92.78)	35.02 (0,72.63)	15.53 (0,39.62)	5.25 (0,16.36)	0 (0,6.5)
LA	20.48 (1.29,36.19)	56.12 (2.97,63.46)	89.72 (0,100)	69.32 (0,96.84)	38.93 (0,78.81)	23.04 (0,64.7)	15.22 (0,51.58)
AVN	12.1 (1.28,23.26)	17.81 (1.49,32.9)	78.95 (0,100)	54.91 (0,100)	19.69 (0,99.94)	0 (0,2.44)	0 (0,0.33)
LAD	9.17 (2.42,11.23)	16.8 (5.27,27.68)	63.66 (1.05,99.53)	35.68 (7.51,76.3)	0.81 (0,17.6)	0 (0,7.63)	0 (0,0.16)
RV	8.84 (2.3,16.4)	32.26 (11.05,41.52)	57.21 (13.74,92.23)	32.42 (0.04,70.1)	1.02 (0.31,27.27)	0.16 (0,5.25)	0 (0,0.09)
RA	9.0 (1.65,18.08)	34.03 (8.83,50.97)	65.44 (0.37,95.73)	24.96 (0.03,71.8)	4.53 (0,45.83)	0.65 (0,7.02)	0 (0,2.04)
SAN	12.54 (2.42,26.25)	24.19 (3.65,35.55)	95.55 (35.39,100)	53.86 (0,100)	5.68 (0,98.39)	0 (0,19.49)	0 (0,15.1)
RCA	8.88 (2.18,16.55)	16.69 (5.0,27.76)	71.67(47.64,100)	32.25(0,96.15)	0(0,7.64)	0(0,7.55)	0(0,0)

### Incidence of acute subclinical myocardial strain alterations

Immediately post-radiotherapy, conventional markers (LVEF, FAC, and TAPSE) showed no detectable decline (all *P* > 0.05). However, the incidence of acute subclinical myocardial strain alterations was high: 40% (16/40) for the left ventricle (LVGLS relative decline ≥ 15%) and 65% (26/40) for the right ventricle (RVFWLS relative decline ≥ 15%), demonstrating enhanced radiosensitivity within the right-sided cardiac system during the hyper-acute phase (Table 3, 5, 6).

**Table 5:** Univariate analysis of factors associated with left ventricular sub-dysfunction.

Variable	*N*	LV-GLS decline≥15% (%)	*P*	Variable	*N*	LV-GLS decline ≥15% (%)	*P*
Age			0.64	LV-*V*_5_			<0.001*
≥60 years	20	38.20		≥46.33%	22	45.70	
<60 years	20	38.90		<46.33%	18	28.60	
Framingham			0.17	LV-*V_10_*			0.042*
≥10	26	37.80		≥29.8%	22	40.40	
<10	14	40.20		<29.8%	18	37.10	
Chemotherapy combined			<0.001*	LV-*V*_20_			0.042*
Yes	16	47.60		≥8.06%	22	40.40	
No	24	33.30		<8.06%	18	37.10	
Immunotherapy combined			<0.001*	AVN *D*_mean_			<0.001*
Yes	8	48.90		≥6.83 Gy	22	45.80	
No	32	35.90		<6.83 Gy	18	28.50	
MHD			<0.001*	AVN-*V*_5_			<0.001*
≥18.08 Gy	18	55.80		≥38.77%	22	45.80	
<18.08 Gy	22	22.90		<38.77%	18	28.50	
Heart-*V*_5_			<0.001*	AVN-*V*_10_			<0.001*
≥70%	18	54.10		≥26.65%	22	45.80	
<70%	22	23.50		<26.65%	18	28.50	
Heart- *V*_10_			<0.001*	AVN-*V*_20_			<0.001*
≥60%	20	49.40		≥16.17%	16	51.50	
<60%	20	25.90		<16.17%	24	29.40	
Heart- *V*_20_			<0.001*	LAD *D*_mean_			<0.001*
≥48.07%	30	57.60		≥10 Gy	18	45.30	
<48.07%	10	31.30		<10 Gy	22	32.60	
Heart-*V*_30_			<0.001*	LAD- *V*_5_			<0.001*
≥21.27%	18	56.50		≥51.17%	22	45.70	
<21.27%	22	22.60		<51.17%	18	28.60	
Heart-*V*_40_			<0.001*	LAD- *V*_10_			<0.001*
≥10.72%	18	56.50		≥36.82%	18	58.30	
<10.72%	22	22.60		<36.82%	22	22.00	
LV *D*_mean_			<0.001*	LAD- *V*_20_			<0.001*
≥16 Gy	16	50.0		≥11.95%	12	40.50	
<16 Gy	24	30.20		<11.95%	28	34.00	

“*” Indicates variables significant results after FDR correction.

**Table 6:** Univariate analysis of factors associated with right ventricular sub-dysfunction.

Variable	*N*	RVFWLS decline≥15% (%)	*P*	Variable	*N*	RVFWLS decline ≥15% (%)	*P*
Age			<0.001*	RV- *V*_5_			<0.001*
≥60 years	20	62.70		≥43.49%	22	76.8	
<60 years	20	68.10		<43.49%	18	56.5	
Framingha			<0.001*	RV-V *V_10_*			<0.001*
≥10	26	81.60		≥17.25%	22	74.2	
<10	14	58.00		<17.25%	18	59.4	
Chemotherapy combined			<0.001*	RV- *V*_20_			<0.001*
Yes	16	85.50		≥7.46%	18	71.7	
No	24	53.30		<7.46%	22	57.2	
Immunotherapy combined			<0.001*	SAN *D*_max_			<0.001*
Yes	8	76.80		≥33.36 Gy	14	69.2	
No	32	62.10		<33.36Gy	26	57.1	
MHD			<0.001*	SAN *D*_mean_			<0.001*
≥18.08 Gy	18	81.40		≥27.9Gy	8	67.80	
<18.08Gy	22	46.80		<27.9Gy	32	55.40	
Heart- *V*_5_			<0.001*	SAN- *V*_5_			<0.001*
≥70%	18	80.90		≥77.23%	20	79.2	
<70%	22	48.50		<77.23%	20	52.5	
Heart- *V*_10_			<0.001*	SAN- *V*_10_			<0.001*
≥60%	20	79.0		≥37%	20	79.2	
<60%	20	53.0		<37%	20	52.5	
Heart- *V*_20_			0.02*	RCA *D*_mean_			<0.001*
≥48.07%	10	65.30		≥6.45Gy	16	69.20	
<48.07%	30	64.30		<6.45Gy	24	57.10	
Heart- *V*_30_			<0.001*	RCA- *V*_5_			<0.001*
≥21.27%	18	72.20		≥77.56%	18	73.0	
<21.27%	22	56.30		<77.56%	22	55.0	
Heart- *V*_40_			<0.001*	RCA- *V*_10_			0.02*
≥10.72%	18	72.20		≥28%	20	68.5	
<10.72%	22	56.30		<28%	20	61.9	
RV- *D*_mean_			<0.001*				
≥11.03Gy	24	73.7					
<11.03Gy	36	53.0					

“*” Indicates variables significant results after FDR correction.

### Quantitative association and risk stratification

Multivariable logistic regression demonstrated that the dichotomized dosimetric metrics of specific substructures were independent predictors of acute strain alterations, exhibiting a pronounced threshold effect based on Maxstat cutoffs (Table 7):

- **AVN:** An AVN *D*_mean_ ≥ 6.83 Gy stratified a significantly higher incidence of LV strain alterations compared to the lower dose group (58.3% vs. 12.5%; *OR*: 4.49, 95% CI: 3.46–5.82, *P* < 0.001).
- **SAN:** A SAN *D*_max_ ≥33.36 Gy significantly accelerated RV strain risks compared to its counterpart (80.8% vs. 35.7%; *OR* = 3.07, 95% CI: 2.11–4.44, *P* < 0.001).
- **RCA:** An RCA *V*_5_ ≥ 77.56% provided the strongest risk stratification, with the incidence of RV strain alterations surging from 7.1% in the low-dose group to 96.2% in the high-dose group (*OR* = 6.03, 95% *CI*: 4.26–8.53, *P* < 0.001).

**Table 7:** Dosimetric thresholds of cardiac sub-structures and their association with reduced LVGLS and RVFWLS.

Dependent variable	Independent predictor	Low-dose group incidence	High-dose group incidence	Coefficient	*OR* (95%*CI*)	*P*
LVGLS	AVN *D*_mean_ ≥6.83Gy	12.5%	58.3%	1.501	4.49 (3.46–5.82)	<0.001
RVFWLS	SAN *D*_max_ ≥33.36Gy	35.7%	80.8%	1.12	3.07 (2.11–4.44)	<0.001
	RCA *V*_5_ ≥ 77.56%	7.1%	96.2%	1.80	6.03 (4.26–8.53)	<0.001

The Spearman correlation matrix confirmed strong spatial collinearity among candidate variables (*r* = 0.71–0.96，all *P* < 0.001); see Supplementary Tables S2 and S3).

## Discussion

Two-dimensional speckle-tracking echocardiography performed 1–2 days post-radiotherapy revealed that the incidence of hyper-acute subclinical myocardial strain alterations far exceeded the detection rates of conventional metrics like LVEF and FAC, with the right-sided cardiac structures exhibiting enhanced susceptibility compared to the left (65% vs. 40%). This confirms that the heart is not a radiologically homogenous organ but possesses pronounced spatial radiosensitivity heterogeneity. Utilizing the Maxstat algorithm, we identified exploratory dosimetric risk cutoffs for SAN *D*_max_, RCA *V_5_* and AVN *D*_mean_, providing preliminary spatial dosimetric reference for refined cardio protection. Given this hyper-early post-RT assessment window, the observed strain reductions likely reflect transient acute microvascular edema or subclinical inflammatory cascades rather than irreversible fibrotic remodeling. Consequently, these ultra-early alterations should be interpreted as early risk warning signals to initiate baseline-referenced, individualized longitudinal dynamic surveillance.

### Sensitivity of 2D-STE and rationale for baseline-referenced surveillance

Post-radiotherapy, conventional LVEF remained within normal limits despite a high incidence of acute subclinical myocardial strain alterations (LVGLS: 40%, RVFWLS: 65%), confirming that 2D-STE outperforms traditional geometric volumetric metrics in identifying localized, early myocardial deformation (1, 14, 15). Notably, although the cohort-wide mean values of RVFWLS showed not statistically significant pre- to post-RT changes, the threshold-based incidence of strain alterations reached 65% when evaluated via a ≥ 15% relative reduction from each patient's baseline. This juxtaposition underscores that cross-sectional evaluations of cohort means are easily neutralized or “masked” by individual physiological compensation (16). Given the wide inter-individual distribution of baseline strain values (17), establishing a longitudinal, patient-specific surveillance architecture referenced against baseline parameters possesses far greater clinical utility than horizontal cohort comparisons, strongly aligning with current international guideline consensus (3, 4, 18).

### Key dosimetric driver of LV alterations: central role of the AVN

Multivariable regression identified an AVN *D*_mean_ ≥ 6.83 Gy as an independent predictor of acute subclinical left ventricular strain alterations. As the central electrophysiological relay station (13), radiation injury to the AVN triggers microstructural specialized myocardial cell damage and interstitial fibrosis, disrupting electrical propagation symmetry (19). This electromechanical uncoupling drives localized mechanical desynchrony within the ventricular wall, manifesting as an early decline in GLS (20, 21). Although LV *D*_mean_ and LAD dosimetric parameters were significant in univariable analyses, their prognostic value was statistically overshadowed by the AVN dose in the multivariable architecture. This phenomenon implies that a structure-specific sparing strategy targeting the conduction network during the hyper-acute phase may offer superior cardio protection compared to conventional whole-heart or myocardial sparing (22, 23). When the AVN threshold is exceeded, patients remain at high risk for strain alterations even if the overall MHD is preserved within safe limits. Notably, due to severe spatial collinearity *(r* = 0.948) and strict events-per-variable constraints, the downstream His bundle was excluded from the final multivariable model (see Supplementary Table S1 and Figure S1).

### Mechanistic drivers of RV radiosensitivity: roles of the SAN and RCA

Multivariable predictors for acute RV strain alterations—SAN *D*_max_ ≥33.36 Gy and RCA *V_5_* ≥ 77.56%—highlight the distinct anatomical and physical dose susceptibility of the right heart. Due to its superficial anterior positioning, the SAN is highly vulnerable to localized high-dose hot spots (*D*_max_), which induce pacing dysregulation and drive downstream hyper-acute electromechanical dyssynchrony (24, 25). This aligns with findings by Atkins et al. (26) linking high right atrial exposure to tachyarrhythmias. Concurrently, because the RCA runs superficially along the atrioventricular groove, it frequently bathes in the low-dose washout or scatter regions of IMRT fields. This diffuse low-dose volume irradiation (V₅) triggers endothelial swelling and microvascular capillary barriers, exerting a significantly heavier pathogenic weight on the thin-walled, low-pressure RV than on the left ventricle (13, 24).

### Synergistic toxicity of multimodal combined regimens

A significantly heightened incidence of acute strain alterations was observed in patients receiving concurrent systemic therapies. The underlying mechanism involves a synergistic biological cascade combining reactive oxygen species (ROS) mediated toxicity from chemotherapy (e.g., anthracyclines, platinum agents) or immune-mediated inflammatory responses from immune checkpoint inhibitors (ICIs) with radiation-induced microvascular endothelial damage (27–29). In modern clinical oncology practice, patients undergoing these combined modalities whose cardiac substructure exposure approaches the exploratory thresholds identified here demand a more intensive, frequent dynamic monitoring schedule (30, 31).

### Exploratory sparing thresholds and limitations

Our data suggest that maintaining an AVN *D*_mean_ < 6.83Gy, SAN *D*_max_ < 33.36Gy, RCA *V*_5_ < 77.56% can serve as exploratory quantitative reference indicators for treatment plan optimization, which can be protected by optimizing multileaf collimator (MLC) trajectories using volumetric modulated arc therapy (VMAT) (32).

Nevertheless, several key limitations must be acknowledged. First, the single-center design and modest sample size placed strict events-per-variable (EPV) constraints on our multivariable regression architectures, preventing the adequate accommodation of potential clinical confounders. Second, the heterogeneous cohort comprised multiple disease sites with differing target volumes and systemic regimens; yet the statistical power was insufficient to support disease-specific subgroup analyses. Consequently, all derived dosimetric cutoffs must be interpreted strictly as exploratory and hypothesis-generating rather than definitive planning constraints. Their clinical safety and generalizability warrant external validation in larger, homogeneous multicenter prospective cohorts with long-term dynamic follow-up.

## Conclusion

Hyper-acute subclinical myocardial strain alterations are highly prevalent immediately following thoracic radiotherapy, with the right-sided cardiac structures exhibiting enhanced acute radiosensitivity. 2D-STE successfully captures localized myocardial biomechanical anomalies prior to any detectable decline in conventional LVEF. Dosimetric metrics of the AVN, SAN, and RCA serve as independent predictors of these early strain alterations. Although these data-driven cutoffs are exploratory and hypothesis-generating rather than definitive planning constraints, they highlight the clinical rationale for structure-specific sparing of the conduction system and right coronary vasculature in precision radiation oncology. Their safety and generalizability warrant independent external validation in larger, homogeneous multicenter prospective cohorts combined with long-term dynamic follow-up for major adverse cardiac events (MACE).

## Data Availability

The original contributions presented in the study are included in the article/Supplementary Material, further inquiries can be directed to the corresponding author.

## References

[1] Walker V Lairez O Fondard O Pathak A Pinel B Chevelle C. Early detection of subclinical left ventricular dysfunction after breast cancer radiation therapy using speckle-tracking echocardiography: association between cardiac exposure and longitudinal strain reduction (BACCARAT study). Radiat Oncol. (2019) 14(1):204. 10.1186/s13014-019-1408-8 31727075 PMC6854785

[2] Bergom C Bradley JA Ng AK Samson P Robinson C Lopez-Mattei J. Past, present, and future of radiation-induced cardiotoxicity: refinements in targeting, surveillance, and risk stratification. JACC CardioOncol. (2021) 3(3):343–59. 10.1016/j.jaccao.2021.06.007 34604796 PMC8463722

[3] Čelutkienė J Pudil R López-Fernández T Grapsa J Nihoyannopoulos P Bergler-Klein J. Role of cardiovascular imaging in cancer patients receiving cardiotoxic therapies: a position statement on behalf of the Heart Failure Association (HFA), the European Association of Cardiovascular Imaging (EACVI) and the Cardio-Oncology Council of the European Society of Cardiology (ESC). Eur J Heart Fail. (2020) 22(9):1504–24. 10.1002/ejhf.1957 32621569

[4] Lyon AR López-Fernández T Couch LS Asteggiano R Aznar MC Bergler-Klein J. 2022 ESC guidelines on cardio-oncology developed in collaboration with the European Hematology Association (EHA), the European Society for Therapeutic Radiology and Oncology (ESTRO) and the International Cardio-Oncology Society (IC-OS). Eur Heart J. (2022) 43(41):4229–361. 10.1093/eurheartj/ehac244 36017568

[5] Integrative Oncology-Cardiology Branch of China Anti-Cancer Association, Ultrasound Medicine Branch of Chinese Medical Association, Chinese Society of Echocardiography. Chinese guideline for the clinical application of noninvasive imaging technology in accessing cancer therapy—related cardiovascular toxicity (2023 edition). Natl Med J China. (2023) 103(42):3367–83. 10.3760/cma.j.cn112137-20230908-00428 37963735

[6] Feat-Vetel J Suffee N Bachelot F Dos Santos M Mougenot N Delage E. X-ray radiotherapy impacts cardiac dysfunction by modulating the sympathetic nervous system and calcium transients. Int J Mol Sci. (2024) 25(17):9483. 10.3390/ijms25179483 39273430 PMC11394929

[7] Bowen Jones S Marchant T Saunderson C McWilliam A Banfill K. Moving beyond mean heart dose: the importance of cardiac substructures in radiation therapy toxicity. J Med Imaging Radiat Oncol. (2024) 68(8):974–86. 10.1111/1754-9485.13737 39228181 PMC11686456

[8] Loap P Kirova Y. Evaluating cardiac substructure radiation exposure in breast rotational intensity modulated radiation therapy: effects of cancer laterality, fractionation and deep inspiration breath-hold. Cancer Radiother. (2021) 25(1):13–20. 10.1016/j.canrad.2020.05.016 33288407

[9] Li X Wu Y Wang Q Li B Wang J. Radiation-induced cardiac substructure damage and dose constraints: a review. Radiat Oncol. (2025) 20(1):94. 10.1186/s13014-025-02668-x 40474260 PMC12139131

[10] Keramida K Farmakis D. Right ventricular involvement in cancer therapy-related cardiotoxicity: the emerging role of strain echocardiography. Heart Fail Rev. (2021) 26(5):1189–93. 10.1007/s10741-020-09938-8 32128669

[11] Feng M Moran JM Koelling T Chughtai A Chan JL Freedman L. Development and validation of a heart atlas to study cardiac exposure to radiation following treatment for breast cancer. Int J Radiat Oncol Biol Phys. (2011) 79(1):10–8. 10.1016/j.ijrobp.2009.10.058 20421148 PMC2937165

[12] Loap P Servois V Dhonneur G Kirov K Fourquet A Kirova Y. A radiation therapy contouring atlas for cardiac conduction node delineation. Pract Radiat Oncol. (2021) 11(4):e434–7. 10.1016/j.prro.2021.02.002 33561549

[13] Zhang Y Han X Chi Z Zhao Z Wang F Liu D. Radiation exposure and clinical validation of autosegmentation models for the supraventricular cardiac conduction system in breast cancer radiotherapy: an institutional perspective. Front Oncol. (2026) 16:1734696. 10.3389/fonc.2026.1734696 41695359 PMC12893944

[14] Li L Jiang X Xie Q. Prognostic value of left ventricular global longitudinal strain on speckle echocardiography for predicting chemotherapy-induced cardiotoxicity in breast cancer patients: a systematic review and meta-analysis. Echocardiography. (2023) 40(4):306–17. 10.1111/echo.15548 36859694

[15] Santoro C Esposito R Lembo M Sorrentino R De Santo I Luciano F. Strain-oriented strategy for guiding cardioprotection initiation of breast cancer patients experiencing cardiac dysfunction. Eur Heart J Cardiovasc Imaging. (2019) 20(12):1345–52. 10.1093/ehjci/jez194 31326981

[16] Espersen C Skaarup KG Lassen MCH Johansen ND Hauser R Olsen FJ. Normal age- and sex-based values of right ventricular free wall and four-chamber longitudinal strain by speckle-tracking echocardiography: from the Copenhagen City Heart Study. Clin Res Cardiol. (2024) 113(3):456–68. 10.1007/s00392-023-02333-x 37968333 PMC10881734

[17] Muraru D Onciul S Peluso D Soriani N Cucchini U Aruta P. Sex- and method-specific reference values for right ventricular strain by 2-dimensional speckle-tracking echocardiography. Circ Cardiovasc Imaging. (2016) 9(2):e003866. 10.1161/CIRCIMAGING.115.003866 26860970

[18] Shi X Wang Y Zhou J. Mechanical property evaluation of the right ventricular myocardium in cancer patients with chemotherapy by echocardiography: a systematic review and meta-analysis. Transl Cancer Res. (2022) 11(5):1122–40. 10.21037/tcr-21-2324 35706806 PMC9189160

[19] Errahmani MY Locquet M Spoor D Jimenez G Camilleri J Bernier M-O. Association between cardiac radiation exposure and the risk of arrhythmia in breast cancer patients treated with radiotherapy: a case-control study. Front Oncol. (2022) 12:892882. 10.3389/fonc.2022.892882 35860581 PMC9289188

[20] Boerma M Hauer-Jensen M. Potential targets for intervention in radiation-induced heart disease. Curr Drug Targets. (2010) 11(11):1405–12. 10.2174/1389450111009011405 20583977 PMC3311026

[21] Tzivoni D Ratzkowski E Biran S Brook JG Stern S. Complete heart block following therapeutic irradiation of the left side of the chest. Chest. (1977) 71(2):231–4. 10.1378/chest.71.2.231 832502

[22] Ell P Martin JM Cehic DA Ngo DTM Sverdlov AL. Cardiotoxicity of radiation therapy: mechanisms, management, and mitigation. Curr Treat Options Oncol. (2021) 22(8):70. 10.1007/s11864-021-00868-7 34110500

[23] Schytte T Hansen O Stolberg-Rohr T Brink C. Cardiac toxicity and radiation dose to the heart in definitive treated non-small cell lung cancer. Acta Oncol. (2010) 49(7):1058–60. 10.3109/0284186X.2010.504736 20831496

[24] Wu SP Tam M Vega RM Perez CA Gerber NK. Effect of breast irradiation on cardiac disease in women enrolled in BCIRG-001 at 10-year follow-up. Int J Radiat Oncol Biol Phys. (2017) 99(3):541–8. 10.1016/j.ijrobp.2017.06.018 29280448

[25] Kim KH Oh J Yang G Lee J Kim J Gwak S- Association of sinoatrial node radiation dose with atrial fibrillation and mortality in patients with lung cancer. JAMA Oncol. (2022) 8(11):1624–34. 10.1001/jamaoncol.2022.4202 36136325 PMC9501754

[26] Atkins KM Zhang SC Kehayias C Guthier C He J Gasho JO. Cardiac substructure radiation dose and associations with tachyarrhythmia and bradyarrhythmia after lung cancer radiotherapy. JACC CardioOncol. (2024) 6(4):544–56. 10.1016/j.jaccao.2024.07.005 39239344 PMC11372031

[27] Gorelik J Vodyanoy I Shevchuk AI Diakonov IA Lab MJ Korchev YE. Esmolol is antiarrhythmic in doxorubicin-induced arrhythmia in cultured cardiomyocytes - determination by novel rapid cardiomyocyte assay. FEBS Lett. (2003) 548(1-3):74–8. 10.1016/S0014-5793(03)00743-9 12885410

[28] Darby SC Cutter DJ Boerma M Constine LS Fajardo LF Kodama K. Radiation-related heart disease: current knowledge and future prospects. Int J Radiat Oncol Biol Phys. (2010) 76(3):656–65. 10.1016/j.ijrobp.2009.09.064 20159360 PMC3910096

[29] Taunk NK Haffty BG Kostis JB Goyal S. Radiation-induced heart disease: pathologic abnormalities and putative mechanisms. Front Oncol. (2015) 5:39. 10.3389/fonc.2015.00039 25741474 PMC4332338

[30] Sun J-Y Qu Q Lou Y-X Hua Y Sun G-Z Sun W. Cardiotoxicity in cancer immune-checkpoint therapy: mechanisms, clinical evidence, and management strategies. Int J Cardiol. (2021) 344:170–8. 10.1016/j.ijcard.2021.09.041 34563597

[31] Hu J-R Florido R Lipson EJ Naidoo J Ardehali R Tocchetti CG. Cardiovascular toxicities associated with immune checkpoint inhibitors. Cardiovasc Res. (2019) 115(5):854–68. 10.1093/cvr/cvz026 30715219 PMC6452314

[32] Pouvreau P Taleb I Fontaine A Edouard L Gibson N Yaouanq M. Heart is a heavy burden: cardiac toxicity in radiation oncology. Support Care Cancer. (2024) 32(11):769. 10.1007/s00520-024-08949-7 39495349

