# Supplementary material for: Beyond mean heart dose: quantitative impact of sub-structure spatial dosimetrics on subclinical myocardial strain in thoracic radiotherapy
Source: Front Cardiovasc Med. 2026 Aug 6;13:1884120. doi: 10.3389/fcvm.2026.1884120 (PMC13489924; doi:10.3389/fcvm.2026.1884120)
Supplement: Supplementary file 1 [file Datasheet4.docx]

| Dose distribution of the sinoatrial node (SAN) in TPS. The SAN was delineated as a sphere (diameter 2.0 cm) tangent to the outer wall of the right atrium, centered at the level of the ascending aorta. Axial, sagittal, and coronal views are shown with Isodose lines (cGy). |  |
| --- | --- |
| Dose distribution of the atrioventricular node (AVN) in TPS. The AVN was delineated as a sphere (diameter 2.0 cm) centered at the junction of the four cardiac chambers, located 1.0 cm above the most inferior slice where the left atrium is visible. Axial, sagittal, and coronal views are shown with Isodose lines (cGy). |  |
| Dose distribution of the bundle of His in TPS. The bundle of His was delineated according to the anatomical landmarks described by Loap et al. (Pract Radiat Oncol, 2021). Axial, sagittal, and coronal views are shown with Isodose lines (cGy). |  |
